# Degradable Controlled Release Fertilizer Composite Prepared via Extrusion: Fabrication, Characterization, and Release Mechanisms

**DOI:** 10.3390/polym12020301

**Published:** 2020-02-02

**Authors:** Siwen Bi, Vincenzo Barinelli, Margaret J. Sobkowicz

**Affiliations:** Department of Plastics Engineering, University of Massachusetts, Lowell, MA 01854, USA; siwen_bi@student.uml.edu (S.B.); vincenzo_barinelli@student.uml.edu (V.B.)

**Keywords:** biodegradable polyesters, controlled release fertilizer, melt blending

## Abstract

In this work, biodegradable polymers were melt compounded with urea phosphate to fabricate “smart fertilizers” for sustainable agriculture. Urea phosphate (UP) is typically applied as a water-soluble fertilizer to treat phosphorus deficiency in high pH soils. Due to the low diffusion rate of phosphate through slow-release fertilizer coatings, phosphate supply has been considered the “bottleneck” for nitrogen–phosphorous–potassium (NPK) nutrients supply. We study the influence of polymer matrix structure on release kinetics in deionized water using novel polyesters including poly (hexamethylene succinate) (PHS), poly (30% butylene succinate-co-70% hexamethylene succinate) (PBHS 30/70), and PBHS 70/30. Melt processed composites of UP and polyester were analyzed to determine UP loading efficiency and dispersion and distribution of the salt in the polymer matrix. A combined empirical model involving diffusion and erosion mechanisms was found have a good agreement with the experimental release curve. This work provides a solution for environmentally friendly controlled release phosphate fertilizer with good release performance using bio-based and biodegradable polymers.

## 1. Introduction

The global consumption of synthetic fertilizer and crop fertilization has steadily increased over the past several decades. For instance, global nitrogen–phosphorus–potassium (NPK) demand was predicted to increase from 135.4 million tons (Mt) in 2000/2001 to 204 Mt in 2023/2024 [1]. However, the fertilization efficiency is still relatively low. Only 50–60% of nitrogen and potassium and 10–25% of phosphorus are estimated to be taken up by crops [2]. Some developments for higher fertilization efficiency have been explored and commercialized. The slow/controlled release of nitrogen fertilizer has been studied over decades to reduce the loss of nitrogen via leaching and evaporation, including urea-aldehyde condensation products, and fertilizer granules/tablets encapsulated with sulfur and/or polymer [3,4,5]. Compared with the use efficiency and the availability of nitrogen, the utility of phosphorous fertilizer should be given more attention. The phosphorus uptake in crops from applied mineral fertilizer reduces dramatically from 15% in the first year to 1–2%/year in subsequent years [3]. Its increasing accumulation in nature due to geochemical sorption, adsorption, and P-precipitation is a major problem termed a “broken biogeochemical cycle”, resulting in the boost of algal biomass, disruption to aquatic ecosystems, and costly recovery [6,7,8,9,10]. Recently, biodegradable polymers as coatings for slow/controlled-release fertilizer have attracted interest for their potential to increase fertilizer/pesticide utilization efficiency and reduce negative environmental effects [5,11,12,13]. However, few studies focus on the controlled release specifically of the phosphate-bearing species and the “bottleneck” problem previously discussed [2,14,15].

The most commonly used commercial high phosphorus fertilizers are triple super phosphate (TSP), di- or mono-ammonium phosphate (DAP/MAP), ammonium polyphosphate (APP), and urea phosphate (UP) [16]. These P fertilizers can have short term effects on the localized pH value in soil; for example, UP, TSP, and MAP have pH values of 1.0–3.5, and DAP has a pH value of 8.0, which could result in “salt burn” of the germinating seed and plant root. Due to high water solubility and good chemical compatibility with N or K fertilizers, P-fertilizers are applied alone or in combination in the form of liquid fertilizer. However, the plant uptake efficiency of liquid fertilizer from foliar feeding is limited [17]. Thus, it would be preferable to apply phosphate to the soil and allow the plant roots to absorb the nutrient. For this, a controlled release method is preferred.

The processing methods for controlled release fertilizers include (1) solvent evaporation-induced phase separation for microcarriers, (2) solution coating on fertilizer granules with/without heat reaction, (3) cold-compressing pellets of fertilizers and binders, and (4) extrusion/melt-blending for polymer/fertilizer composite tablets/films [2,18,19,20,21]. Extrusion or melt blending for the preparation of controlled release nitrogen fertilizers (such as urea and nitrogen inhibitor) is particularly attractive because the process is simple, cheap, and does not involve solvents, high pressure, or reactive mixing [19,20,22,23]. Thermogravimetric analysis combined with Fourier transform infrared spectra can be a good technique to understand the possible thermal decomposition during processing.

Biodegradable polymers are an important component of many controlled release devices in pharmaceutical delivery applications and recently in tissue engineering [24]. Linear polyesters are the most widely-researched class, especially poly(lactic acid) (PLA), poly(glycolic acid) (PGA), and their copolymer, P(LA-*co*-GA) [25,26]. The controlled release kinetics can be classified according to the following release mechanisms: (1) diffusion-controlled, (2) swelling/dissolution-controlled, (3) degradation/erosion-controlled; and (4) osmotically controlled [27]. Sackett et al., Arifin et al., and Versypt et al., reviewed the mathematical modeling and simulations of drug release of biodegradable controlled release systems [25,28,29]. Biodegradable polymers (such as polybutylene succinate (PBS), polyhydroxyalkanoates, biopolyol/methylene diphenyl diisocyanate, and starch) have been applied alone or in combination as encapsulating materials for controlled release fertilizer/pesticide, especially in the form of coated granules [2,19,20,21,30,31]. Irfan et al., reviewed and reported the latest development in modeling and simulation of nutrients release, mainly based on coated fertilizer, but the effects of polymer degradation and external environment (such as temperature of soil) are less studied [32]. In reviewing the literature, we found less information regarding modeling controlled release from biodegradable matrices [19].

Here, controlled release phosphate/polymer composites were prepared using a twin-screw micro-extruder to melt mix urea phosphate with poly (hexamethylene succinate) and its copolyesters with PBS. Our previous work showed that poly (hexamethylene succinate) (PHS) has a similar chemical structure to PBS, a similar crystallinity, and faster biodegradability, while random copolymers of PBS and PHS have tunable thermal, mechanical, and biodegradable properties [33,34]. This work investigates the influence of copolymer structure on extrusion processing and the phosphate release mechanisms of urea phosphate (UP)/polymer composite tablets. Thermogravimetric analysis is used to verify UP loading level and prove that melt processing has not chemically degraded the fertilizer. Elemental analysis is conducted to study the distribution of UP in the polymer matrix during release. The temperature of the release medium is also considered. This work can provide a practical strategy to process eco-friendly controlled release fertilizer and predict the phosphate release depending on polyester characteristics.

## 2. Materials and Methods

### 2.1. Materials

Succinic acid (SA, ≥99%), 1,4-butanediol (BD), 1,6-hexanediol (HD, 97%), and titanium (IV) isopropoxide (TTIP, 97%) were used as received from Sigma-Aldrich (Saint Louis, MO, USA) for the synthesis of PHS, poly(30 mol % butylene succinate-co-70 mol % hexamethylene succinate) (PBHS30/70), and PBHS70/30 via melt polycondensation, and then synthesized polymers were purified via precipitation from chloroform into methanol, as described in our previous work [33,34]. Urea phosphate (UP, ≥ 98%), l-ascorbic acid (AA, reagent grade), and ammonium molybdate (meets the United States Pharmacopeia testing specifications) were provided by Sigma-Aldrich (Saint Louis, MO, USA).

### 2.2. Sample Preparation

Urea phosphate granules in the size range of 600–850 μm were sifted out with a Ro-Tap sieve shaker (from W.S. Tyler, Mentor, OH, USA) and U.S.A standard testing sieves No. 20 and No.30 (from W.S. Tyler, Mentor, OH, USA). UP (0.5 g) and purified polyesters (PHS and PBHS30/70, 4.5 g) were blended in a micro compounder (from DACA Instruments, Santa Barbara, CA, USA) at a screw speed of 50 RPM and temperature of 80 °C for one min. Urea phosphate and PBHS70/30 were blended at the same conditions except at a temperature of 90 °C. Urea phosphate-loaded polyester blends were molded with compression molding in a heated press into tablet samples of Φ 9.4 mm × 1.3 mm at 60 °C for PBHS30/70, 65 °C for PHS, and 90 °C for PBHS70/30.

### 2.3. Characterization

The Fourier transform infrared (FTIR) spectra of UP-loaded polyester tablets were measured in the range of 4000–400 cm^−1^ using a Nicolet iS50 Fourier transform infrared spectrometer (from Thermo Electron Company, Waltham, MA, USA) with a smart orbit attenuated total reflectance (ATR) accessory. The spectra were obtained using 32 scans. The thermal stability of UP, pure polyesters, and UP/polyester blends were measured with a Discovery thermogravimetric analyzer (TGA) (from TA Instruments, New Castle, DE, USA) from room temperature to 1000 °C at the heating rate of 20 °C/min under air atmosphere. The thermal decomposition of UP was determined using a 5500 TGA (from TA Instruments, New Castle, DE, USA) connected with an FTIR module from room temperature to 200 °C at the heating rate of 20 °C/min under nitrogen. The nitrogen flow rate and purge rate were 50 mL/min. The FTIR spectra were collected over 16 scans with the resolution of 4 cm^−1^ (each set of scans took approximately 30 s). The residue from TG-FTIR was collected and tested with ATR-FTIR for the analysis of composition.

PBHS30/70_10% UP blends and PBHS70/30_10% UP blends were dissolved in chloroform at concentration of 50 mg/mL and then cast on glass slides. The morphology of the sample was observed by a microscope (LSCM FV300 from Olympus, Tokyo, Japan). To determine the loading efficiency of blending, samples were dissolved in chloroform at around 1 mg/mL, and phosphate species were extracted by adding 10 mL water and stirring overnight at 650 rpm. The phosphate amount in top water solutions were tested using a DU 640 ultraviolet-visible (UV-Vis) spectrophotometer (from Beckman Coulter, Brea, CA, USA).

UP/polyester blends before and after immersion in deionized water were freeze-fractured with liquid nitrogen. The cross-section surface was coated with gold using a Desk IV vacuum sputter coater (from Denton Vacuum, Moorestown, NJ, USA) and observed with field-emission scanning electron microscope (SEM) (JSM 7401F from JEOL, Peabody, MA, USA). Energy-dispersive spectroscopy (EDS) (from JEOL, Peabody, MA, USA) was used to analyze the change of element distribution on the cross-section of samples at intervals during the release test. The constituents of data analysis included carbon, oxygen, and phosphorous without consideration of gold from sputter coating.

### 2.4. Phosphate Release Characteristics

A UP-loaded polyester tablet was incubated in a glass bottle of 100 mL deionized water and kept in a water bath of 25 °C or 37 °C. Samples of 5 mL were taken at certain intervals for phosphate determination and fresh DI water was added to keep the volume constant. The accumulated release of phosphate in solution samples was measured using the phospho-molybdate colorimetric assay and calibration of the peak in the UV-Vis spectra at 860 nm [35].

## 3. Results

**Figure 1:**
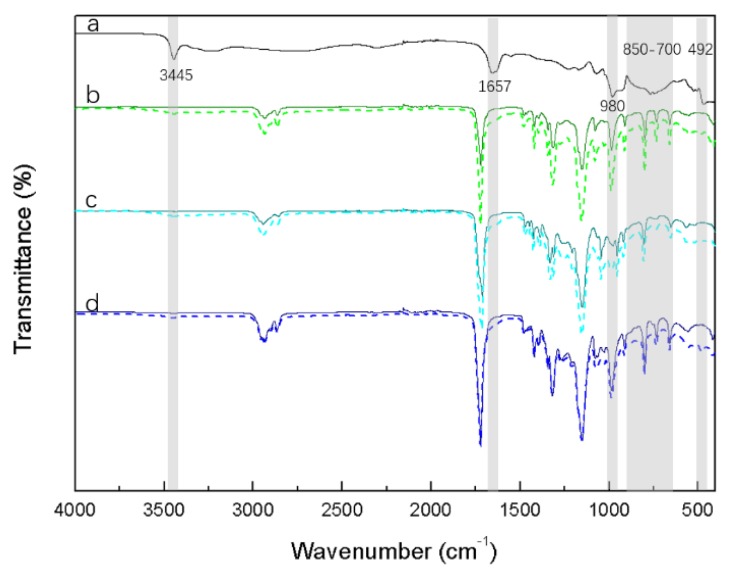
Attenuated total reflectance–Fourier transform infrared (ATR-FTIR) spectra of (**a**) urea phosphate (UP), (**b**) poly (hexamethylene succinate) (PHS) (solid line) and PHS_UP blend (dashed line), (**c**) poly (70% butylene succinate-co-30% hexamethylene succinate) (PBHS 70/30) (solid line) and PBHS 70/30_UP blend (dashed line), and (**d**) poly (30% butylene succinate-co-70% hexamethylene succinate) (PBHS 30/70) (solid line) and PBHS 30/70_UP blend (dashed line).

The polyester–UP composites were investigated using spectroscopy and thermogravimetric analysis to verify the successful incorporation of UP. The ATR-FTIR spectra of UP, neat polyesters (PHS, PBHS30/70, and PBHS70/30) and UP-loaded polyesters are depicted in Figure 1. IR spectra of polyesters show characteristic bands at 2860–2960 cm^−1^, 1720 cm^−1^, 1153 cm^−1^, 800–920 cm^−1^, and 733 cm^−1^, which are attributed to stretching of C–H in methylene groups, the stretching vibration of carbonyl (–C=O) in ester groups, ether (–C–O–C–) stretching vibration, C–C backbone stretching, and the rocking of –(CH_2_)_6_–,respectively [34,36]. After the loading of UP, the transmittance of urea phosphate characteristic peaks, particularly at 1657 cm^−1^, can be seen in the composite spectra. FTIR shows evidence of UP in all blends but cannot provide quantitative loading percentage.

### 3.1. Thermal Decomposition of Urea Phosphate

**Figure 2:**
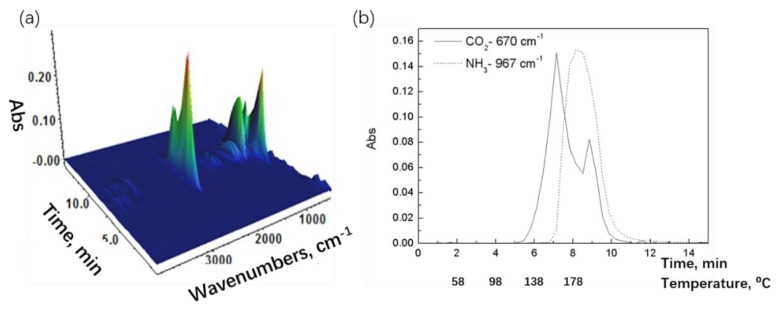
(**a**) Three-dimensional (3D) thermogravimetric (TG)-FTIR spectra and (**b**) absorbance of characteristic peaks vs. time/temperature.

Interaction between urea and phosphate can reduce the ammonia volatilization from urea [37]. Heating and shear in blending processing can also cause the decomposition of UP; in particular, the urea component decomposes over the temperature range 100–200 °C. TG-FTIR analysis was applied to determine the decomposition mechanism and temperature for UP. In Figure 2a, the 3D TG-FTIR spectra show the overall IR absorption of gaseous species produced from the thermal decomposition of UP in the range of 25–200 °C as a function of time. From Figure 2a, the gaseous species were identified, and these are presented in Table 1. The absorption of characteristic peaks for carbon dioxide (CO_2_) and ammonia (NH_3_) as a function of time and temperature in Figure 2b were derived from Figure 2a and TG analysis. Figure 2b shows CO_2_ produced at 5.4 min corresponding to 126 °C and NH_3_ produced at 7.1 min corresponding to 153 °C due to the fixation by phosphoric acid. This result indicates that urea started to decompose into CO_2_ and NH_3_ at about 126 °C, with a small amount of water evolved from the decomposition of phosphoric acid.

**Figure 3:**
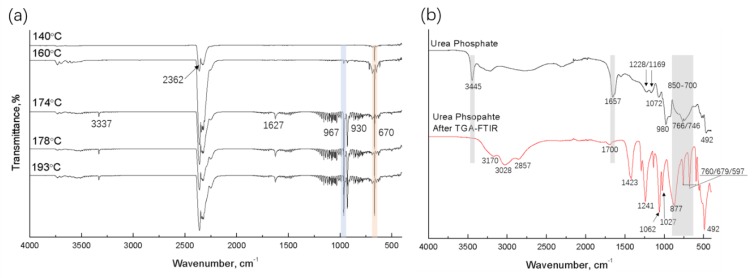
(**a**) FTIR spectra of UP off-gas during thermogravimetric analyzer (TGA) heating at different temperatures and (**b**) ATR-FTIR spectra of UP before and after TG-FTIR.

**Figure 4:**
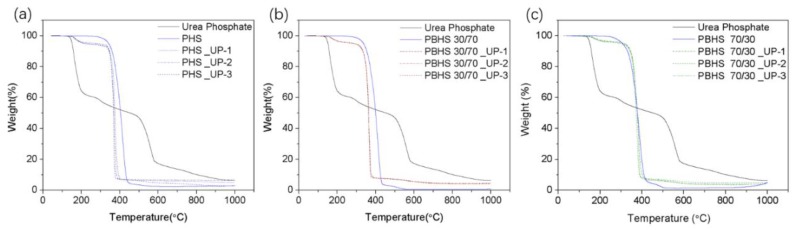
TGA of neat polymer, urea phosphate with (**a**) PHS_UP blends, (**b**) PBHS 30/70_UP blends, and (**c**) PBHS 70/30_UP blends.

In Figure 3b, the single peak of urea phosphate at 3445 cm^−1^ represents a secondary amide (-NH-) group rather than the expected two peaks of free primary amide in urea, which is likely due to the interaction between urea and phosphate. After TG-FTIR, the IR spectrum of UP residue was compared with that of untreated UP in Figure 3b; the assignments of characteristic bands are summarized in Table 2. In Figure 3b, the characteristic bands of the amide group (3445 cm^−1^, 1657 cm^−1^, and 700–850 cm^−1^ broad band) in UP disappeared, indicating that most of the nitrogen and carbon in urea had already been emitted as NH_3_ and CO_2_ [40,41,42]. The dehydration of phosphoric acid mainly occurred after 210 °C and continuously and slowly formed H_4_P_2_O_7_/HPO_3_ until the rapid formation of P_4_O_10_ at 579 °C, as demonstrated by the weight loss of pure UP in Figure 4 [36,40]. From TG-FTIR analysis, we determine that the processing temperature of UP/polymer blends should stay below 126 °C to avoid premature thermal decomposition. Since melt processing can result in overheating through viscous dissipation, excessive shear should also be avoided.

### 3.2. Loading Efficiency

In order to verify the target 10 wt % loading during the blending process, we analyzed the TGA weight loss curve and also performed a dissolution/UV-Vis test. TG analysis of UP, neat polymer, and UP/polymer blends are shown in Figure 4; UP-loaded samples were repeated three times to study the uniformity within the samples produced by melt blending. The loading efficiencies of UP/polymer blends were analyzed by comparing the weight loss of UP/polymer blends and weight loss of UP (38.9%) over the temperature range of 110–210 °C (ignoring the weight loss of polyester) associated with the thermal decomposition of only the urea component (38.9 wt % of the UP compound). The average weight loss of UP-loaded PHS, PBHS 30/70, and PBHS 70/30 are respectively 3.83 ± 0.25%, 3.60 ± 0.04%, and 3.14 ± 0.26% in this range. Then, these values were corrected by the weight fraction of urea in UP to obtain the experimentally determined weight loading for all blends presented in Table 3. The blends were also dissolved in chloroform, and the phosphate was extracted in distilled water. The amount of phosphate extracted was measured using the molybdenum blue/ascorbic acid colorimetric assay; the loading efficiencies thus obtained are also presented in Table 3 [35]. The derived loading levels indicate that there is no loss of UP for PHS and PBHS 30/70 blends, while PBHS 70/30 blends have about 1–2% loss during blending due to a higher processing temperature of 90 °C combined with shear.

### 3.3. Characterization of Blends Morphology

**Figure 5:**
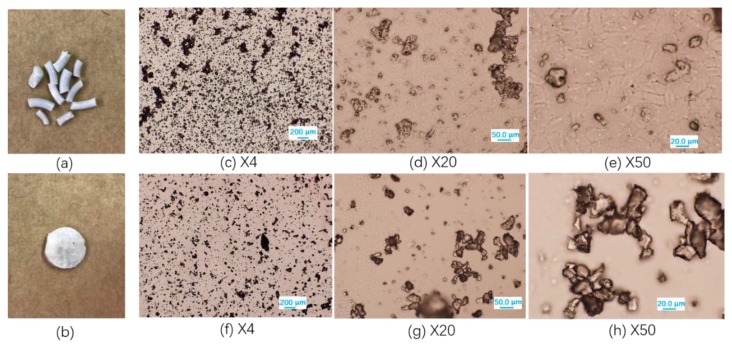
Photos of UP/polymer blends (**a**) from extrusion and (**b**) compression molding; optical microcopy images of urea phosphate crystal after processing (**c**–**e**) for UP-PBHS 30/70 blend; and (**f**–**h**) for UP-PBHS 70/30 blend at different magnification.

The physical appearance of extruded and compressed polyester/UP blends (taking PBHS 70/30_UP blends as an example) is shown in Figure 5a,b. The extruded filament and compressed tablet are light gray due to the added urea phosphate crystals; the solid samples became white after the release of nutrients. In Figure 5c–h, the UP crystal structures were observed using optical microscopy after the dissolution of UP/polymer blends in chloroform. The extrusion process resulted in the decrease of UP crystal size from 600–850 μm to 10–30 μm, and the sequence of feeding also affected the crystal size. UP crystals in PBHS 30/70_UP blends are smaller likely because they were fed at the same time as the polymer pellets. For PBHS 70/30_UP blends, UP was added after polymer pellets were melted; thus, less mechanical shear was imparted on the UP crystals for that blend.

**Figure 6:**
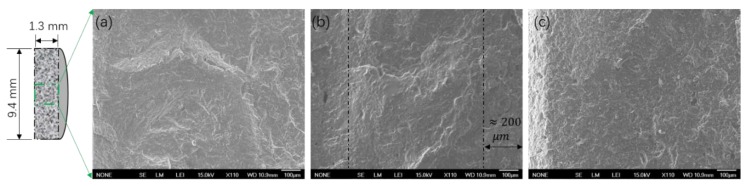
Scanning electron microscope (SEM) images of a cross-sectional surface from a PHS_UP blends tablet (**a**) before release, (**b**) at 72 h, and (**c**) at 588 h in water of 25 °C.

**Figure 7:**
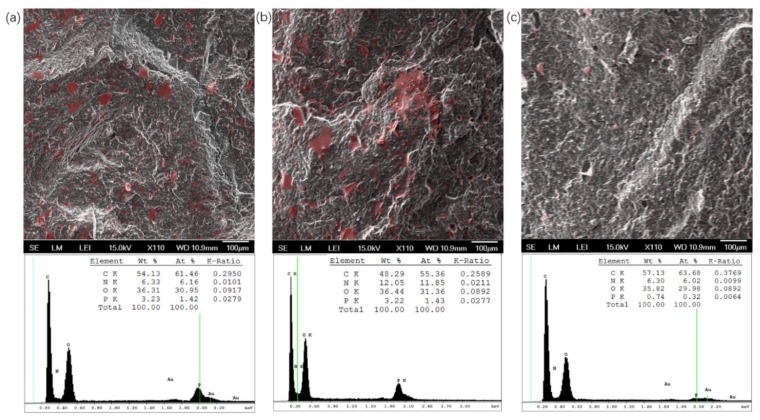
SEM-EDS mapping (top) and element analysis spectra (bottom) of cross-section surface from a 10% UP-loaded PHS tablet (**a**) before release, (**b**) at 72 h, and (**c**) at 588 h in water of 25 °C (red represents locations with phosphorous present). EDS: energy-dispersive spectroscopy.

In Figure 6, the cross-sectional morphology and the distribution of elemental phosphorus in PHS_UP blend tablets were investigated during the nutrients release using SEM-EDS. While it is difficult to accurately quantify the loading using this technique, the EDS analysis in Figure 7a shows that 3.23 wt % of the sample surface is elemental phosphorous. Since phosphorous makes up 19.6 wt % of UP, the value is higher than expected, but it is not unreasonable for the 10 wt % composites. In the first stage of UP release, the samples are immersed in deionized water, and exposed UP crystals are dissolved and released into water, which is very fast due to the high solubility of UP (11.74 mol % at 25 °C and 15.42 mol % at 37 °C) [47]. In addition, the diffusion coefficients of urea and phosphoric acid in water at 25 °C are high (around 1.382 × 10^−5^ cm^2^/s and 0.3–0.9 × 10^−6^ cm^2^/s respectively), which also results in the fast transport of UP into the water [48,49]. As UP dissolved out of the polymer matrix, a porous skin layer of about 200 μm formed, as shown in Figure 6b. In Figure 7b, the remaining elemental phosphorus can be seen in/around pores on the right edge, which indicates that water can access the pore structure deeper in the polymer matrix. As UP crystals dissolve and diffuse into polymer matrix, the areas where the phosphorous appears become larger. The EDS elemental analysis shows that even though about 40% of the UP is released at 72 h (see Section 3.4), the amount of nitrogen and phosphorous visible in the image is more than or similar to that of pre-release blends due to the diffusion of nutrients in the polymer matrix. In Figure 6c and Figure 7c at 588 h, all the crystals break down and diffuse in the polymer matrix or out of the polymer. EDS elemental analysis reveals that compared to the weight percentage of phosphorus in as-prepared blends, about 20% remains in the polymer matrix. The amount of residual phosphate in the matrix could correspond to the solubility of phosphate in polymer.

### 3.4. Phosphate Release and Modeling of Initial Kinetic Behavior

**Figure 8:**
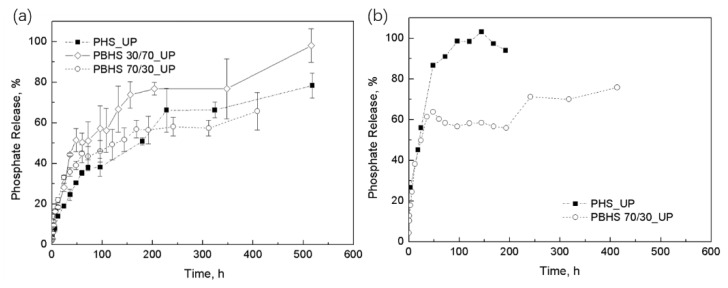
Accumulative phosphate release fraction for (**a**) UP-loaded PHS, PBHS 30/70, and PBHS 70/30 at 25 °C; (**b**) UP-loaded PHS and PBHS 70/30 at 37 °C.

The experimentally determined loading levels (Table 3) were used to calculate the accumulated fractional release of phosphate during immersion in deionized water, as shown in Figure 8a at 25 °C and Figure 8b at 37 °C. During the release testing, no visible swelling was observed. After one hour of release at 25 °C, about 5–10% of total phosphate was mobilized in water from the exposed UP crystals. In Figure 8a, the first-release equilibrium (linear trend established) of PBHS 30/70_UP, PBHS 70/30_UP, and PHS_UP occurs at 48 h, 60 h, and 72 h, respectively. After that, the release rate slows for all samples. The release of PBHS 70/30_UP levels off at around 60%, while the release continues to around 75% (PBHS 30/70_UP) and 65% (PHS_UP), followed by a continuous release stage. In Figure 8b, the release of PBHS 70/30 at 37 °C appears similar to that at 25 °C but reaches 60% release faster (about 48 h versus 72 h at 25 °C), while the release of PHS_UP at 37 °C shows much faster release until the entire UP payload is released within about 100 h.

The Korsmeyer-Peppas model [50,51] is described by the following equation:

(1) $$\frac{M_{t}}{M_{\infty }}=k_{1}t^{n}$$

where *M_t_* is the release phosphate at time *t*, *M*_∞_ is the loading of phosphate in a polymer matrix, *n* is the release exponent, and *k* is the kinetic constant.

**Figure 9:**
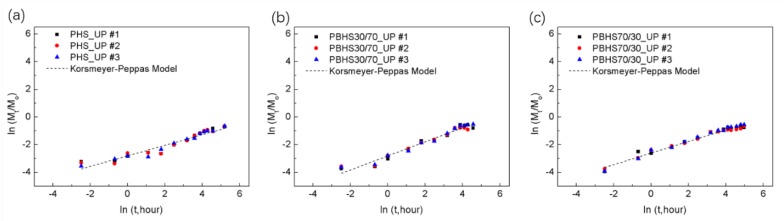
Simulation of initial release at 25 °C. (**a**) PHS_UP samples; (**b**) PBHS30/70_UP samples; (**c**) PBHS70/30_UP samples.

The Korsmeyer-Peppas model (*t* in hour) for controlled release was applied to fit the polymer/UP tablet release behavior up to *M_t_*/*M*_∞_< 0.6 in Figure 9; the results are summarized in Table 4. In the Korsmeyer–Peppas model, *n* is < 0.45 for cylindrical tablets corresponding to the Fickian diffusion model, while 0.45 < *n* < 0.89 indicates non-Fickian transport. Our polyester/UP blends (PHS_UP and PBHS 70/30_UP in Figure 9a,c) exhibit Fickian diffusion except for the polymer with majority butylene monomer, the PBHS 30/70_UP tablet in Figure 9b, which trends toward non-Fickian transport, as indicated by the higher exponent of 0.51. This composite is also well described by the Higuchi model, which fixes the exponent at 0.5. This result is comparable with the release of nitrification inhibitor (dicyandiamide) slow release pellets (*n* < 0.45) and that of herbicide (metribuzin) slow release pellets (*n* = 0.49) published by Levett’s group and Boyandin’s group [19,52]. According to the *R*^2^ values, most tablet release curves have better agreement with the Korsmeyer-Peppas model. The Korsmeyer-Peppas model only demonstrates diffusion-controlled release, indicating that in the first 60% release for all tablets, diffusion transport dominates [53].

### 3.5. Discussion and Modeling of Diffusion-Degradation Release Behavior

**Figure 10:**
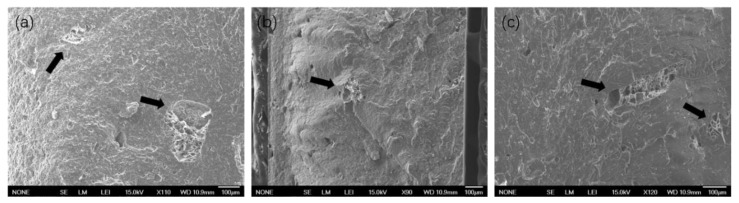
SEM spectra of degradation structure in (**a**) PHS_UP for 588-h release, (**b**) PBHS 30/70_UP for 606-h release, and (**c**) PBHS 70/30_UP for 580-h release at 25 °C.

PHS and PBHS polyesters are hydrophilic, and the carbonyl groups in aliphatic polyesters are susceptible to hydrolytic degradation in acid/alkali environments [54]. Polyesters are known for water absorption in storage; poly(butylene succinate) can absorb about 1% water over 60 days of immersion, resulting in a reduction of mechanical and thermal properties [55]. The pH of UP solution is about 2, and the diffusion of urea is faster than that of phosphate, which could result in a localized decrease of pH and acceleration of hydrolytic degradation in the pores as UP dissolves. These effects should lead to early degradation in the UP solution. Figure 10 shows areas of the composite where degradation is evident around larger crystal structures after release, which also occurs in poly(lactic-co-glycolic acid) drug delivery systems with autocatalysis reported by Siepmann’s group and Mylonaki’s group [56,57]. EDS elemental analysis indicates locally higher phosphate concentration in the degraded pore structures. These phenomena demonstrate that lots of phosphate can be trapped in aggregates, which can explain the leveling-off and burst regions in the release curves. This can also happen in a coated controlled release system, causing stalling in nutrients release.

In order to describe the release behavior over the entire length of the release experiment, we examined the curve fitness of experimental results with two different models: the diffusion-relaxation model (Equation (2)) and a model that combines diffusion and erosion (Equation (3)) [58,59].

(2) $$\frac{M_{t}}{M_{\infty }}=k_{1}t^{m}+k_{2}t^{2m}$$

where *m* is the diffusional exponent of 0.4625, $k_{1}$ is associated with diffusion, and $k_{2}$ is associated with relaxation.

(3) $$\frac{M_{t}}{M_{\infty }}=at^{0.5}+bt+ct^{2}+dt^{3}$$

where *a* is associated with diffusion, and *b*, *c*, and *d* are associated with erosion.

The simulated release for all samples was determined using the diffusion–relaxation model and the diffusion–erosion model in Figure S1 and Figure S2 (in Supplementary Materials). In Table 5, the relaxation constants for all the tablets reveal a minimal relaxation effect on diffusion (i.e., *k*_2_ is small; therefore, there is no second-order time dependence). With the consideration of erosion in the matrix, the empirical model has good agreement with the experimental release in Table 5. Since *b*, *c*, and *d* are all much smaller than *a*, we can see that diffusion dominates for this model as well. Although some values are negative, it does appear that the contributions from *b*, *c*, and *d* are larger for the copolymers than for pure PHS, which correlates well with their degradation rates (PHS is the slowest degrading polymer of those tested here) [33]. Perhaps small corrections to late-stage release rates can be accounted for in this empirical model, but additional experimental work is needed to test the fits to these models more robustly.

Phosphate release tends to be slower than other nutrients and shows a leveling-off around 45–70% [2]. In our system, the good dispersion of UP crystals in the tablets can provide a solution to this problem, as shown in the release of PHS_UP and PBHS 30/70_UP. While the ratio of comonomers did not have a strong influence on the release rate in water, their processing temperatures are quite different, which can influence the particle size and UP degradation during processing. The copolyesters also have different degradation behavior in enzymatic environments, which could influence the release rate in a real setting [33]. Furthermore, our results show the influence of temperature. At 37 °C, the faster diffusion of water and nutrients can accelerate the dissolution and reduce the trapping of nutrients, resulting in a shorter level-off stage in the release curve.

## 4. Conclusions

In this study, we proposed a reliable method to extrude urea phosphate/polymer composites of high loading efficiency for environmentally friendly biodegradable controlled release fertilizer using PHS and PBHS copolyesters. The loading levels from dissolution/UV-Vis (based on phosphate) and TGA (based on urea) have good agreement. From the analysis of FTIR and optical microscopy, we can conclude that urea phosphate was dispersed from the initial size of 600–850 μm to 10–30 μm during processing. From the mathematical simulation, we can conclude that the relaxation due to water uptake did not influence the release, and the erosion–diffusion model had a good match with the release behavior of all the tablets. However, the erosion–diffusion model did not consider the influence of variable nutrients concentration on matrix erosion, which should be investigated in future work, especially for different sized crystals dispersed in a polymer matrix. Last but not the least, we found that the smaller crystals dispersed in a matrix can be a promising solution to phosphate bottlenecks in controlled release fertilizer in agriculture application and even in other controlled release fields.

## Figures and Tables

**Table 1:** Identification of major gaseous species through characteristic IR bands.

Gaseous Species	Characteristic IR Bands (cm^−1^)	Ref.
**CO_2_**	2362, 670	[38,39]
**NH_3_**	3337, 1627, 967, 930	[36,38]
**H_2_O (minor)**	4000–3500, 1850–1640, 1560–1400	[38]

**Table 2:** Assignment of FTIR absorption bands of urea phosphate before and after TG-FTIR.

$\bar{\upsilon },\;{\mathrm{cm}}^{-1}$	Assignment	Ref.	$\bar{\upsilon },\;{\mathrm{cm}}^{-1}$	Assignment	Ref.
Urea Phosphate		-	Urea Phosphate after TGA-FTIR		-
3445	N-H stretching in secondary amide	[40]	3200–3600	O-H vibration (broad band)	[40]
			3170	N-H vibration (free/bonded)	[40]
3218 (broad)	O-H/N-H valence vibration	[43]	3028, 2857	C-H stretching/deformation	[36,41]
1650–1690	C=O stretching (amide I band)	[40]	1241, 1295	P=O stretching vibration	[44]
1160–1260	P-O stretching	[42]	1062, 1027	P-O stretching vibration (P-O-P or P-O-C)	[45]
970–1010 1050–1140	P=O for ROPO_3_^2-^	[42,46]	877	Typical of bicyclic O=PO_3_ structure	[42]
700–850 (broad)	N-H bending/wagging	[41,42]	760, 679	Typical of cyclic phosphate structure	[42]
492	O=P-OH	[42]	492	O=P-OH	[42]

**Table 3:** Composite weight loading from dissolution/UV-Vis test and TGA.

Blends	Feed Ratio, %	UP Loading, % From UV-Vis	UP Loading, % From TGA
PHS_UP	10	9.9 ± 0.1	9.9 ± 0.6
PBHS 30/70_UP	10	10.4 ± 0.5	9.3 ± 0.1
PBHS 70/30_UP	10	7.8 ± 0. 4	8.1 ± 0.7

**Table 4:** Simulation using Korsmeyer-Peppas model.

Blends	Korsmeyer–Peppas Model			
	*n*	*k*_1_	*R* ^2^	Type of Diffusion
25 °C	-			
PHS_UP	0.38 ± 0.01	0.060 ± 0.002	0.94 ± 0.02	Fickian
PBHS 30/70_UP	0.51 ± 0.01	0.060 ± 0.002	0.963 ± 0.006	Non-Fickian
PBHS 70/30_UP	0.42 ± 0.02	0.074 ± 0.002	0.98 ± 0.009	Fickian
37 °C	-			
PHS_UP	0.3333	0.001838	0.9752	Fickian
PBHS 70/30_ UP	0.4227	0.125093	0.9914	Fickian

**Table 5:** Simulation using diffusion-relaxation model and erosion–diffusion model.

Blends	Diffusion–Relaxation Model			Erosion–Diffusion Model				
	$k_{1}$	$k_{2}$	*R* ^2^	a	b	c	d	*R* ^2^
25 °C	-							
PHS_UP	0.053 ± 0.001	−0.0005 ± 0.0003	0.97 ± 0.01	0.038 ± 0.003	0.0008 ± 0.0003	−3 × 10^−6^ ± 1 × 10^−6^	−3 × 10^−9^ ± 2 × 10^−9^	0.98 ± 0.01
PBHS 30/70_UP	0.09 ± 0.01	−0.0021 ± 0.0006	0.94 ± 0.04	0.059 ± 0.006	0.0016 ± 0.0009	−1.4 × 10^−5^ ± 6 × 10^−6^	−1.9 × 10^−8^ ± 9 × 10^−9^	0.97 ± 0.01
PBHS 70/30_UP	0.077 ± 0.007	−0.0024 ± 0.0003	0.973 ± 0.09	0.0595 ± 0.005	7 × 10^−26^ ± 1 × 10^−25^	−1.1 × 10^−5^ ± 2 × 10^−6^	−1 × 10^−9^ ± 1 × 10^−9^	0.97 ± 0.02
37 °C	-							
PHS_UP	0.18144	−0.00819	0.93165	0.11544	0.00181	4.12 × 10^−5^	7.5 × 10^−8^	0.96772
PBHS 70/30_ UP	0.11332	−0.00458	0.85088	0.15763	−0.012	2.4 × 10^−6^	−2.3 × 10^−8^	0.96073

## Supplementary Materials

The following are available online at https://www.mdpi.com/2073-4360/12/2/301/s1, Figure S1: Simulation using diffusion-relaxation model. Figure S2: Simulation using diffusion–erosion model.

## References

[1] Heffer P., Prud M. Fertilizer Outlook 2019–2023. Proceedings of the 87th IFA Annual Conference.

[2] Lubkowski K., Smorowska A., Grzmil B., Kozłowska A. (2015). Controlled-release fertilizer prepared using a biodegradable aliphatic copolyester of poly (butylene succinate) and dimerized fatty acid. J. Agric. Food. Chem..

[3] Trenkel M.E. (2010). Slow-and Controlled-Release and Stabilized Fertilizers: An Option for Enhancing Nutrient Use Efficiency in Agriculture.

[4] Naz M.Y., Sulaiman S.A. (2016). Slow release coating remedy for nitrogen loss from conventional urea: A review. J. Control. Release.

[5] Naz M.Y., Sulaiman S.A. (2017). Attributes of natural and synthetic materials pertaining to slow-release urea coating industry. Rev. Chem. Eng..

[6] Gu Y.-J., Han C.-L., Fan J.-W., Shi X.-P., Kong M., Shi X.-Y., Siddique K.H., Zhao Y.-Y., Li F.-M. (2018). Alfalfa forage yield, soil water and P availability in response to plastic film mulch and P fertilization in a semiarid environment. Field Crop. Res..

[7] Dawson C., Hilton J. (2011). Fertiliser availability in a resource-limited world: Production and recycling of nitrogen and phosphorus. Food Policy.

[8] Cordell D., Drangert J.O., White S. (2009). The story of phosphorus: Global food security and food for thought. Global Environ. Change.

[9] Scholz R.W., Ulrich A.E., Eilittä M., Roy A. (2013). Sustainable use of phosphorus: A finite resource. Sci. Total. Environ..

[10] Elser J., Bennett E. (2011). Phosphorus cycle: A broken biogeochemical cycle. Nature.

[11] Calabi-Floody M., Medina J., Rumpel C., Condron L.M., Hernandez M., Dumont M., de la Luz Mora M. (2018). Smart fertilizers as a strategy for sustainable agriculture, in Advances in Agronomy. Adv. Agron..

[12] Campos E.V.R., De Oliveira J.L., Fraceto L.F. (2014). Applications of Controlled Release Systems for Fungicides, Herbicides, Acaricides, Nutrients, and Plant Growth Hormones: A Review. Adv. Sci. Eng. Med..

[13] Chen J., Lü S., Zhang Z., Zhao X., Li X., Ning P., Liu M. (2018). Environmentally friendly fertilizers: A review of materials used and their effects on the environment. Sci. Total. Environ..

[14] Yao Y., Gao B., Chen J., Yang L. (2013). Engineered Biochar Reclaiming Phosphate from Aqueous Solutions: Mechanisms and Potential Application as a Slow-Release Fertilizer. Environ. Sci. Technol..

[15] Broschat T.K., Moore K.K. (2007). Release Rates of Ammonium-Nitrogen, Nitrate-Nitrogen, Phosphorus, Potassium, Magnesium, Iron, and Manganese from Seven Controlled-Release Fertilizers. Commun. Soil Sci. Plant Anal..

[16] McCauley A., Jones C., Jacobsen J. (2009). Commercial fertilizers and soil amendments. Nutrient Management—A Self-Study Course from MSU Extension Continuing Education Series.

[17] Totten F.W., Liu H., Mccarty L.B., Baldwin C.M., Bielenberg D.G., Toler J.E. (2008). Efficiency of Foliar Versus Granular Fertilization: A Field Study of Creeping Bentgrass Performance. J. Plant Nutr..

[18] Liu L., Shen T., Yang Y., Gao B., Li Y.C., Xie J., Tang Y., Zhang S., Wang Z., Chen J. (2018). Bio-based Large Tablet Controlled-Release Urea: Synthesis, Characterization, and Controlled-Released Mechanisms. J. Agric. Food Chem..

[19] Levett I., Pratt S., Donose B.C., Brackin R., Pratt C., Redding M.R., Laycock B. (2019). Understanding the Mobilization of a Nitrification Inhibitor from Novel Slow Release Pellets, Fabricated through Extrusion Processing with PHBV Biopolymer. J. Agric. Food Chem..

[20] Hongsriphan N., Pinpueng A. (2019). Properties of Agricultural Films Prepared from Biodegradable Poly (Butylene Succinate) Adding Natural Sorbent and Fertilizer. J. Polym. Environ..

[21] Liu Y., Guo B.-H. (2019). Preparation and characterisation of poly(butylene succinate) microcarriers containing pesticide. Micro Nano Lett..

[22] Xiao X., Yu L., Xie F., Bao X., Liu H., Ji Z., Chen L., Xie D.F. (2017). One-step method to prepare starch-based superabsorbent polymer for slow release of fertilizer. Chem. Eng. J..

[23] Liu T.G., Wang Y.T., Guo J., Liu T.B., Wang X., Li B. (2017). One-step synthesis of corn starch urea based acrylate superabsorbents. J. Appl. Polym. Sci..

[24] Burg A.J.L., Orr D.E., Buchanan F.J. (2008). An overview of bioresorbable materials. Degradation Rate of Bioresorbable Materials: Prediction and Evaluation. Synthetic Bioresorbable Polymers.

[25] Versypt A.N., Pack D.W., Braatz R.D. (2013). Mathematical modeling of drug delivery from autocatalytically degradable PLGA microspheres—A review. J. Control. Release.

[26] Makadia H.K., Siegel S.J. (2011). Poly Lactic-co-Glycolic Acid (PLGA) as Biodegradable Controlled Drug Delivery Carrier. Polymers.

[27] Peppas N.A., Narasimhan B. (2014). Mathematical models in drug delivery: How modeling has shaped the way we design new drug delivery systems. J. Control. Release.

[28] Arifin D.Y., Lee L.Y., Wang C.-H. (2006). Mathematical modeling and simulation of drug release from microspheres: Implications to drug delivery systems. Adv. Drug Deliv. Rev..

[29] Sackett C.K., Narasimhan B. (2011). Mathematical modeling of polymer erosion: Consequences for drug delivery. Int. J. Pharm..

[30] Fertahi S., Bertrand I., Amjoud M., Oukarroum A., Arji M., Barakat A. (2019). Properties of Coated Slow-Release Triple Superphosphate (TSP) Fertilizers Based on Lignin and Carrageenan Formulations. ACS Sustain. Chem. Eng..

[31] Wu C.-S. (2008). Controlled release evaluation of bacterial fertilizer using polymer composites as matrix. J. Control. Release.

[32] Irfan S.A., Razali R., KuShaari K., Mansor N., Azeem B., Versypt A.N.F. (2018). A review of mathematical modeling and simulation of controlled-release fertilizers. J. Control. Release.

[33] Bi S., Tan B., Soule J.L., Sobkowicz M.J. (2018). Enzymatic degradation of poly (butylene succinate-co-hexamethylene succinate). Polym. Degrad. Stab..

[34] Tan B., Bi S., Emery K., Sobkowicz M.J. (2017). Bio-based poly(butylene succinate-co-hexamethylene succinate) copolyesters with tunable thermal and mechanical properties. Eur. Polym. J..

[35] Nagul E.A., McKelvie I.D., Worsfold P., Kolev S.D. (2015). The molybdenum blue reaction for the determination of orthophosphate revisited: Opening the black box. Anal. Chim. Acta.

[36] Marcilla A., Beltran M.I., Gómez-Siurana A., Martinez-Castellanos I., Berenguer D., Pastor V., García A.N. (2015). TGA/FTIR study of the pyrolysis of diammonium hydrogen phosphate–tobacco mixtures. J. Anal. Appl. Pyrolysis.

[37] Fan M.X., MacKenzie A.F. (1993). Urea and Phosphate Interactions in Fertilizer Microsites: Ammonia Volatilization and pH Changes. Soil Sci. Soc. Am. J..

[38] Wang Z., Lv P., Hu Y., Hu K. (2009). Thermal degradation study of intumescent flame retardants by TG and FTIR: Melamine phosphate and its mixture with pentaerythritol. J. Anal. Appl. Pyrolysis.

[39] Xia Z., Singh A., Kiratitanavit W., Mosurkal R., Kumar J., Nagarajan R. (2015). Unraveling the mechanism of thermal and thermo-oxidative degradation of tannic acid. Thermochim. Acta.

[40] Saihi D., Vroman I., Giraud S., Bourbigot S. (2005). Microencapsulation of ammonium phosphate with a polyurethane shell part I: Coacervation technique. React. Funct. Polym..

[41] Colthup N.B., Daly L.H., Wiberley S.E. (2012). Introduction to Infrared and Raman Spectroscopy.

[42] Balabanovich A. (2005). Thermal decomposition study of intumescent additives: Pentaerythritol phosphate and its blend with melamine phosphate. Thermochim. Acta.

[43] Abdel-Kader A., Ammar A., Saleh S. (1991). Thermal behaviour of ammonium dihydrogen phosphate crystals in the temperature range 25–600° C. Thermochim. Acta.

[44] Pretsch E., Buehlmann P., Affolter C., Pretsch E., Bhuhlmann P., Affolter C. (2000). Structure Determination of Organic Compounds.

[45] Jiang W., Hao J., Han Z. (2012). Study on the thermal degradation of mixtures of ammonium polyphosphate and a novel caged bicyclic phosphate and their flame retardant effect in polypropylene. Polym. Degrad. Stab..

[46] Wang A., Liu N., Yin H., Wu H., Wada Y., Ren M., Jiang T., Cheng X., Xu Y. (2007). Size-controlled synthesis of hydroxyapatite nanorods by chemical precipitation in the presence of organic modifiers. Mater. Sci. Eng. C.

[47] Tang J.W., Mu R.Z., Zhang B.L., Fan X.S. (2007). Solubility of urea phosphate in water+ phosphoric acid from (277.00 to 354.50) K. J. Chem. Eng. Data.

[48] Ruiz-Beviá F., Fernández-Sempere J., Boluda-Botella N. (1995). Variation of phosphoric acid diffusion coefficient with concentration. AIChE J..

[49] Winkelmann J., Lechner M.D. (2017). Diffusion coefficient of urea in water. Diffusion in Gases, Liquids and Electrolytes.

[50] Higuchi T. (1961). Rate of Release of Medicaments from Ointment Bases Containing Drugs in Suspension. J. Pharm. Sci..

[51] Dash S., Murthy P.N., Nath L., Chowdhury P. (2010). Kinetic modeling on drug release from controlled drug delivery systems. Acta Pol. Pharm. Drug Res..

[52] Boyandin A.N., Zhila N.O., Kiselev E.G., Volova T.G. (2016). Constructing Slow-Release Formulations of Metribuzin Based on Degradable Poly(3-hydroxybutyrate). J. Agric. Food Chem..

[53] Siepmann J., Siepmann F. (2008). Mathematical modeling of drug delivery. Int. J. Pharm..

[54] Jung J.H., Ree M., Kim H. (2006). Acid- and base-catalyzed hydrolyses of aliphatic polycarbonates and polyesters. Catal. Today.

[55] Lee J.M., Ishak Z.M., Taib R.M., Law T.T., Thirmizir M.A. (2013). Mechanical, thermal and water absorption properties of kenaf-fiber-based polypropylene and poly (butylene succinate) composites. J. Poly. Environ..

[56] Siepmann J., Elkharraz K., Siepmann F., Klose D. (2005). How Autocatalysis Accelerates Drug Release from PLGA-Based Microparticles: A Quantitative Treatment. Biomacromolecules.

[57] Mylonaki I., Allémann E., Delie F., Jordan O. (2018). Imaging the porous structure in the core of degrading PLGA microparticles: The effect of molecular weight. J. Control. Release.

[58] Upadrashta S.M., Katikaneni P.R., Hileman G.A., Keshary P.R. (1993). Direct Compression Controlled Release Tablets Using Ethylcellulose Matrices. Drug Dev. Ind. Pharm..

[59] Peppas N.A., Sahlin J.J. (1989). A simple equation for the description of solute release. III. Coupling of diffusion and relaxation. Int. J. Pharm..

