# Supplementary material for: Degradable Controlled Release Fertilizer Composite Prepared via Extrusion: Fabrication, Characterization, and Release Mechanisms
Source: Polymers (Basel). 2020 Feb 2;12(2):301. doi: 10.3390/polym12020301 (PMC7077398; doi:10.3390/polym12020301)
Supplement: Supplementary file 1 [file polymers-12-00301-s001.pdf]

Degradable Controlled Release Fertilizer Composite Prepared via Extrusion: Fabrication, Characterization, and Release Mechanisms

Siwen Bi, Vincenzo Barinelli and Margaret J. Sobkowicz

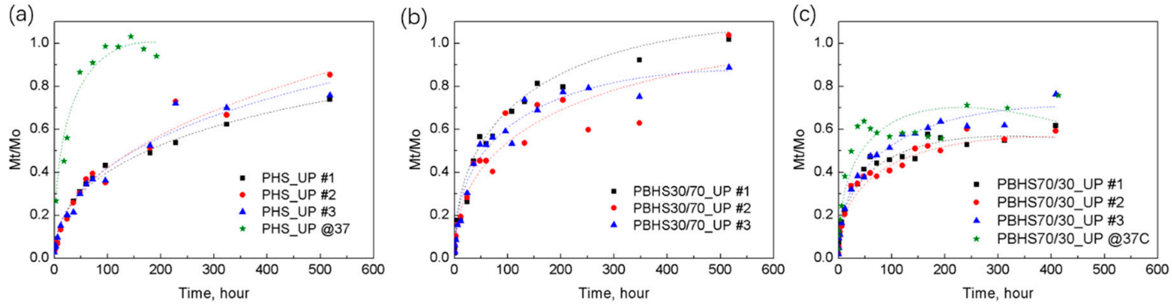

Figure S1. Simulation using diffusion-relaxation model.

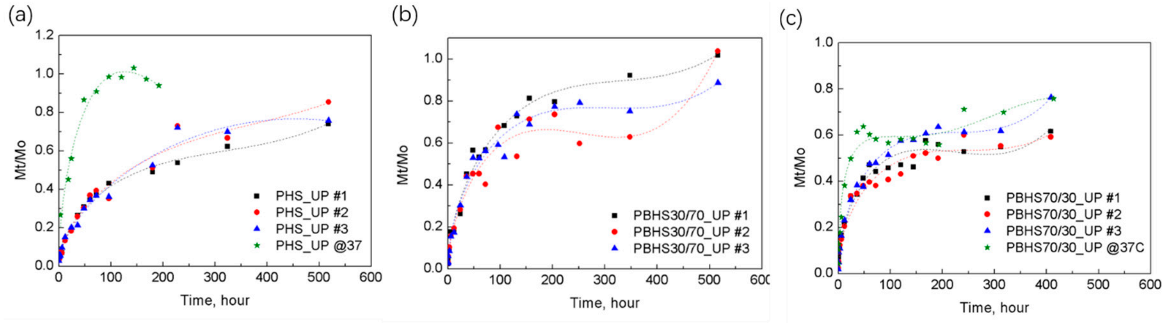

Figure S2. Simulation using diffusion-erosion model.

In order to describe the release behavior over the entire length of the release experiment, we examined the curve fitness of experimental results with two different models: (1) the diffusion-relaxation model ($\frac{M_t}{M_\infty} = k_1 t^m + k_2 t^{2m}$, m is the diffusional exponent of 0.4625) and (2) a model that combines diffusion and erosion ($\frac{M_t}{M_\infty} = at^{0.5} + bt + ct^2 + dt^3$). The release of all samples at 25 °C and 37 °C was simulated using the diffusion-relaxation model in Figure S1 and the diffusion-erosion model in Figure S2.
